# Glucose/Xylose Co-Fermenting *Saccharomyces cerevisiae* Increases the Production of Acetyl-CoA Derived n-Butanol From Lignocellulosic Biomass

**DOI:** 10.3389/fbioe.2022.826787

**Published:** 2022-02-16

**Authors:** Yeon-Jung Lee, Phuong Hoang Nguyen Tran, Ja Kyong Ko, Gyeongtaek Gong, Youngsoon Um, Sung Ok Han, Sun-Mi Lee

**Affiliations:** ^1^ Clean Energy Research Center, Korea Institute of Science and Technology (KIST), Seoul, South Korea; ^2^ Department of Biotechnology, Korea University, Seoul, South Korea; ^3^ Division of Energy and Environment Technology, University of Science and Technology (UST), Daejeon, South Korea; ^4^ Green School, Korea University, Seoul, South Korea

**Keywords:** *Saccharomyces cerevisiae*, glucose/xylose co-fermentation, n-butanol, acetyl-CoA, acetate, lignocellulosic biomass

## Abstract

Efficient xylose catabolism in engineered *Saccharomyces cerevisiae* enables more economical lignocellulosic biorefinery with improved production yields per unit of biomass. Yet, the product profile of glucose/xylose co-fermenting *S. cerevisiae* is mainly limited to bioethanol and a few other chemicals. Here, we introduced an n-butanol-biosynthesis pathway into a glucose/xylose co-fermenting *S. cerevisiae* strain (XUSEA) to evaluate its potential on the production of acetyl-CoA derived products. Higher n-butanol production of glucose/xylose co-fermenting strain was explained by the transcriptomic landscape, which revealed strongly increased acetyl-CoA and NADPH pools when compared to a glucose fermenting wild-type strain. The acetate supplementation expected to support acetyl-CoA pool further increased n-butanol production, which was also validated during the fermentation of lignocellulosic hydrolysates containing acetate. Our findings imply the feasibility of lignocellulosic biorefinery for producing fuels and chemicals derived from a key intermediate of acetyl-CoA through glucose/xylose co-fermentation.

## Introduction

Lignocellulosic biomass offers a sustainable and environmentally friendly source of raw materials for producing fuels and chemicals (Ko and Lee 2018). Commercial bioethanol production has been achieved using the yeast *Saccharomyces cerevisiae* and sugar- or starch-based biomass. To improve the economic feasibility of lignocellulosic biorefinery, *S. cerevisiae* strains have been engineered to co-ferment glucose and xylose, the most abundant hexose and pentose sugars in lignocellulosic hydrolysates, respectively, resulting in significantly improved lignocellulosic bioethanol yields, titers, and productivity (Hoang Nguyen Tran et al., 2020). In addition, increasing efforts have also been devoted to expanding the product profile of *S. cerevisiae* to include advanced fuels and chemicals demonstrating the great potential of microbial cell factories for biorefinery (Ekas et al., 2019). Yet, the production of non-native products using both glucose and xylose derived from lignocellulosic hydrolysates has been less explored. Thus, it is necessary to evaluate the feasibility of sustainable-biorefinery concepts based on lignocellulosic biomass, which is the most abundant and sustainable resource.

Efficient glucose/xylose co-fermentation enables complete utilization of all available sugars in lignocellulosic biomass, which increases the overall conversion yield in lignocellulosic biorefinery. By incorporating xylose into the substrate used by *S. cerevisiae*, the overall conversion yield could be increased by up to 84% during lignocellulosic bioethanol production (Ko et al., 2018). In addition, xylose metabolism supported the diversion of metabolic flux towards non-ethanol products in an ethanol-producing *S. cerevisiae* yeast strain (Li et al., 2019). Recently, xylose-utilizing strains have been suggested as promising microbial cell factories for producing acetyl-CoA-derived products (Kwak and Jin 2017), due to their altered cellular metabolism. Therefore, it is of interest to confirm the advantages of glucose/xylose co-fermentation in producing fuels and chemicals derived from acetyl-CoA at the phenotypic and transcriptional levels.

As a core intermediate in central carbon metabolism, acetyl-CoA can be converted into various fuels and chemicals. Yeast strains have been suggested to be better hosts for producing acetyl-CoA-derived products than other microbial hosts (Sun and Alper 2020). With limited metabolic flux through cytosolic acetyl-CoA, however, the successful production of fuels and chemicals derived from acetyl-CoA has been rarely demonstrated in *S. cerevisiae*. Specifically, the production of acetyl-CoA-derived n-butanol, a short-chain alcohol similar to ethanol, has been limited even with higher butanol tolerance of *S. cerevisiae* when compared to other non-native bacterial hosts (Knoshaug and Zhang 2009; Hong and Nielsen 2012; González-Ramos et al., 2013). Engineering *S. cerevisiae* for n-butanol production has been based mainly on the reverse-β-oxidation pathway of *Clostridium sp.*, which naturally produce butanol. Therefore, successful n-butanol production would also enable successful incorporation of the reverse-β-oxidation pathway, an economic route for medium-chain fatty acids and alcohols with numerous industrial applications, into the cellular network of *S. cerevisiae.*

Here, we sought to evaluate the potential of a glucose/xylose co-fermenting strain of *S. cerevisiae* as a production host for acetyl-CoA-derived n-butanol in lignocellulosic biorefinery concept. To this end, an n-butanol-biosynthesis pathway was introduced in XUSEA, a previously developed *S. cerevisiae* strain that simultaneously ferments glucose and xylose (Tran Nguyen Hoang et al., 2018; Hoang Nguyen Tran et al., 2020), generating XUSEA-B. The transcription profile of the XUSEA strain was compared to that of the wild-type strain to understand the modified cellular network caused by the introduction of a heterologous route for bioconverting xylose into butanol. n-Butanol production in the glucose/xylose co-fermenting strain was further improved by supplementation with acetic acid, a major by-product generated during lignocellulosic biomass-pretreatment process. Finally, we successfully demonstrated lignocellulosic n-butanol production by using a glucose/xylose co-fermenting strain. Consequently, the results of this study show the potential of a glucose/xylose co-fermenting strain in the production of acetyl-CoA-derived n-butanol from lignocellulosic biomass.

## Materials and Methods

### Strains and Culture Conditions

All strains used in this study are shown in Table 1. The yeast strains used in this study were isogenic with respect to *S. cerevisiae* S288C BY4741. The yeast strains were routinely cultivated at 30 °C in yeast synthetic complete (YSC) medium composed of xylose (and/or glucose), 6.7 g/L of yeast nitrogen base (Difco, Detroit, MI, United States), and CSM-HIS-LEU-URA (MP Biomedicals, Solon, Ohio, United States). *Escherichia coli* DH10β cells were employed for DNA manipulation and were cultured at 37°C in LB medium supplemented with 100 μg/ml ampicillin.

**TABLE 1:** Strains used in this study.

Strain	Description	References
XUSEA	BY4741 gre3 xylA*3 TAL1 XKS1 RPE1 Δgre3 Δpho13 Δasc1 evolved on xylose	Hoang Nguyen Tran et al. (2020)
XUSAE57	BY4741 xylA*3 TAL1 XKS1 RPE1 Δgre3 Δpho13 evolved on xylose and acetic acid stress	Ko et al. (2019)
XUSAE-B	XUSAE57 p423-*Hbd*-*Crt*, p425-*Erg10*-_cyto_ *Etr1*, and p426-*BdhB-EutE*	This study
XUSAEA-B	XUSAE-B *acs* ^L641P^ from *Salmonella enterica* and *aadh* from *E. coli*	
WT-B	BY4741 p423-*Hbd*-*Crt*, p425-*Erg10*-_cyto_ *Etr1*, and p426-*BdhB-EutE*	
XUSEA-B (XA-4)	XUSEA p423-*Hbd*-*Crt*, P425-*Erg10*-_cyto_ *Etr1*, and p426-*BdhB-EutE*	
XA -1	XUSEA p423-_cyto_ *Kr*-_cyto_ *Htd*, p425-*Erg10*-_cyto_ *Etr1*, and p426-*AdhE2*-*EutE*	
XA -2	XUSEA p423-_cyto_ *Kr*-_cyto_ *Htd*, p425-*Erg10*-_cyto_ *Etr1*, and p426-*BdhB*-*EutE*	
XA -3	XUSEA p423-*Hbd*-*Crt*, p425-*Erg10*-_cyto_ *Etr1*, and p426-*AdhE2*-*EutE*	
XA -5	XUSEA p423-_cyto_ *Kr*-_cyto_ *Htd*, P425-*Erg10*-_cyto_ *Etr1*, and p426-*AdhE2*	
XA -6	XUSEA p423-*Hbd*-*Crt*, p425-*Erg10*-_cyto_ *Etr1*, and p426-*AdhE2*	

### Plasmid Construction

All plasmids used in this study are shown in Table 2. The homologous genes used in this study were *ERG10* and *ETR1*, and the heterologous genes were *EutE* from *E. coli*, *Kr* and *Htd* from *Yarrowia lipolytica*, and *Hbd*, *Crt*, *AdhE2*, and *BdhB* from *Clostridium acetobutylicum*. The start codon of *BdhB* was changed from GTG to ATG, and the mitochondria-targeting sequences of the Kr, Htd, and Etr1 proteins were predicted and excluded using TargetP software version 1.1 (http://www.cbs.dtu.dk/services/TargetP/) and the MITOPROT online tools (https://ihg.gsf.de/ihg/mitoprot.html) according to the reference by Lian and Zhao (2015). Each gene cassette was cloned into an expression vector using the Gibson Assembly method and introduced in *S. cerevisiae* using the Frozen-EZ Yeast Transformation II Kit (Zymo Research), according to the manufacturer’s instructions.

**TABLE 2:** Plasmids used in this study.

Plasmid	Characteristics
p423-_cyto_ *Kr*-_cyto_ *Htd*	GPDp_-cyto_ *Kr*-PRM9t and TEFp-_cyto_ *Htd*-CPS1t
p423-*Hbd-Crt*	GPDp-*Hbd-*PRM9t and TEFp*-Crt*-CPS1t
p423-*Hbd-Crt-Ter*	GPDp-*Hbd-*PRM9t, TEFp*-Crt*-CPS1t, and HXT7p*-Ter*-TPI7t
p423-*Hbd-Crt-Ter* ^G155C^	GPDp-*Hbd-*PRM9t, TEFp*-Crt*-CPS1t, and HXT7p*-Ter* ^G155C^-TPI7t
p423-*Hbd-Crt-*_cyto_ *Etr1*	GPDp-*Hbd-*PRM9t, TEFp*-Crt*-CPS1t, and HXT7p*-*_cyto_ *Etr1*-TPI7t
p425-*Erg10-*_cyto_ *Etr1*	TEFp-*Erg10-*CYC1t and HXT7p*-*_cyto_ *Etr1*-TPI7t
p426-*AdhE2*	CYC1p-*AdhE2-*SPG5t
p426-*AdhE2-EutE*	CYC1p-*AdhE2-*SPG5t and PGK1p*-EutE*-CYC1t
p426-*BdhB*	CYC1p-*BdhB-*SPG5t
p426*-BdhB-EutE*	CYC1p-*BdhB-*SPG5t and PGK1p*-EutE*-CYC1t
p426-*BdhB-EutE-Erg10*	CYC1p-*BdhB-*SPG5t, PGK1p*-EutE*-CYC1t, and TEFp-*Erg10-*CYC1t

### Predicting Mitochondria-Targeting Sequences

The online MITOPROT tool was used to predict and exclude mitochondria-targeting signal peptide sequences, which were further validated using TargetP, version 1.1 (Lian and Zhao 2015). The nucleotide sequences encoding the excluded signal peptides are as follows: TTC​CGA​CTC​ACC​ACT​GCC​CGA​ATT​GCT​TCT​GTG​CGA​GGCTT​CTC​CAC​CTC​CGC​CAG​CCT​GTC​C (for *Kr*), CGA​AGC​CTA​TAT​ATA​AAC​GTT CCG​GGT​CTT​TTT​CCT​TCC​ACC​TCT​CTA​GCA​CGA​GAA (for *Htd*), and CTTCCCA CATTCAAACGTTACATG (for *Etr1*). Additionally, an intron sequence of *Htd* was excluded along with mitochondria-targeting sequence.

### Fermentation

For seed cultures, yeast cells from a glycerol stock were inoculated in YSC medium containing 2% glucose. The yeast cells were then transferred to fresh YSC medium containing 2% glucose, 2% xylose, or 2% glucose plus 2% xylose with an inoculum size of 5%, and grown aerobically in flasks for 1.5–2 days. Subsequently, the yeast cells were harvested and finally transferred to fresh YSC medium. The pH of the fermentation medium was maintained by adding 70 mM phthalate buffer (pH 5.0) or 80 mM phosphate buffer (pH 6.5). Micro-aerobic fermentation was carried out in 125 ml serum bottles with a final working volume of 40 ml at a low cell density (initial optical density [OD] of 0.2) and a high cell density (initial OD of 15). The serum bottles were capped with rubber stoppers, with a needle for carbon dioxide release during fermentation. Lignocellulosic-biomass hydrolysates (*Miscanthus sacchariflorus Goedae-Uksae 1*), pretreated with dilute acid, were purchased from Sugaren Co., Ltd (Korea). The hydrolysates contained 30.6 g/L of glucose, 15.1 g/L of xylose, 1.3 g/L of arabinose, 0.15 g/L of formic acid, 0.05 g/L of acetic acid, 0.08 g/L of levulinic acid, 0.06 g/L of 5-HMF, and 0.07 g/L of furfural.

### Analytical Methods

Cell growth was analyzed by measuring the OD at 600 nm with a Cary 60 Bio UV–Vis spectrometer (Agilent Technologies, United States). Glucose and xylose concentrations were analyzed using a high-performance liquid chromatography system (HPLC 1260 Infinity, Agilent Technologies, CA, United States) equipped with a refractive index detector, using a Hi-Plex H column (Agilent Technologies, Palo Alto, CA, United States). The system was operated using 5 mM H_2_SO_4_ as the mobile phase at a flow rate of 0.6 ml/min, and the column temperature was maintained at 65 °C. Ethanol, n-butanol, and acetate concentrations were analyzed using a gas chromatograph instrument (Agilent Technologies, CA, United States) equipped with a flame ionization detector, using an HP-INNOWax polyethylene glycol column (30 m × 0.32 µm × 0.25 µm).

### Transcriptomic Analysis

For transcriptomic analysis, cells were cultured and harvested at exponential phase during glucose fermentation and glucose/xylose co-fermentation. Cell pellets were collected by centrifugation at 1,000 *g* for 5 min. Total RNA extraction was performed using Trizol reagent (Invitrogen, CA, United States) according to the manufacturer’s protocol provided by Ebiogen (Seoul, Republic of Korea). Each of the total RNA samples was evaluated for RNA quality control based on the 28S/18S ratio and RIN measured on the 2,100 Bioanalyzer system (Agilent Technologies, Waldbronn, Germany). The cDNA library was constructed using the Clontech SMARTer Stranded RNA-Seq kit (Clontech, Mountain View, CA, United States). High-throughput sequencing was performed on an Illumina HiSeq 2,500 system (Illumina, Inc, San Diego, CA, United States).

## Results

### Introduction of a n-Butanol Production Pathway in a Glucose/Xylose Co-fermenting Strain

**FIGURE 1:**
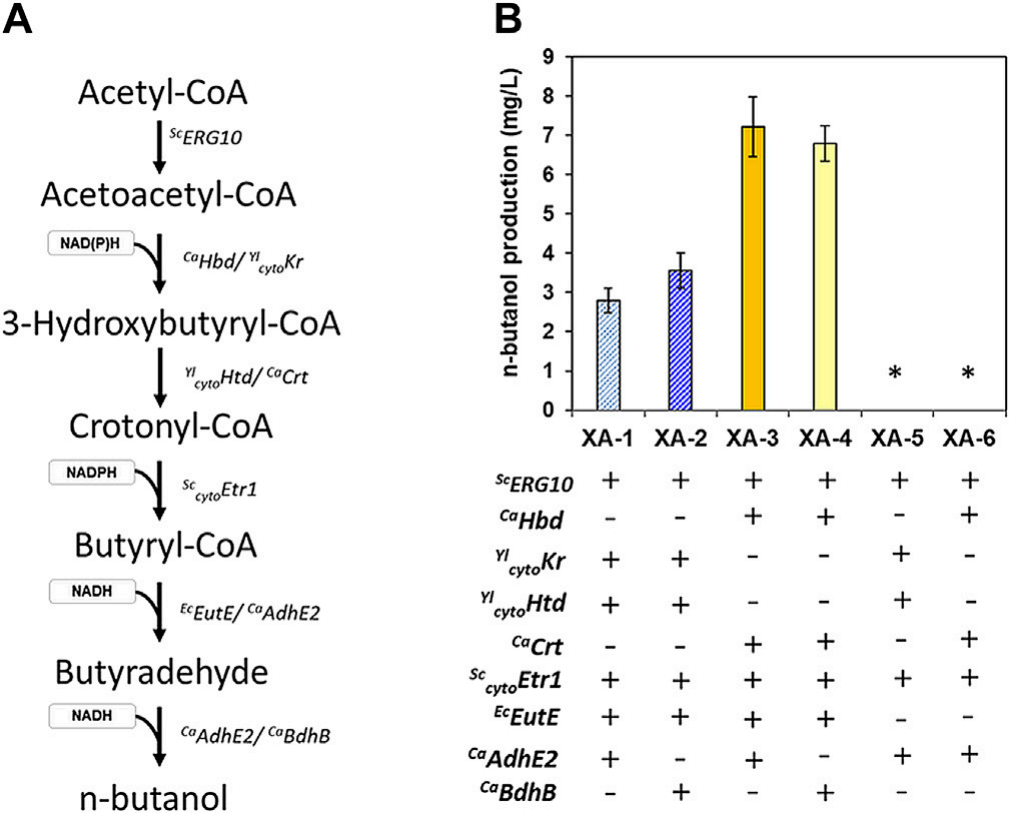
n-butanol production in the glucose/xylose co-fermenting *S. cerevisiae* strains expressing various gene sets used for n-butanol biosynthesis. The genes were sourced from *S. cerevisiae* (*Sc*), *Y. lipolytica* (*Yl*), *E. coli* (*Ec*), and *C. acetobutyricum* (*Ca*). Low-cell density fermentations (initial OD of 0.2) were conducted with 20 g/L xylose as the sole carbon source. **(A)** n-butanol pathway showing the genes used in this study. **(B)** butanol titer of the strains expressing six combinations of genes used to construct various n-butanol-production pathways. The error bars represent the standard deviations obtained using biological triplicates. *: below the detection limit of 2.5 mg/L.

To develop an *S. cerevisiae* strain capable of n-butanol-production, genes that were previously reported to support butanol biosynthesis were introduced in an efficient glucose/xylose co-fermenting strain, XUSEA (Hoang Nguyen Tran et al., 2020), with various combinations. Specifically, *Hbd* and *Crt* originated from *C. acetobutylicum* (a representative n-butanol-producing bacterium), and the orthologous genes (*Kr* and *Htd*, respectively) originated from *Y. lipolytica*, a yeast with an efficient *β*-oxidation pathway. These genes were heterologously expressed along with the homologous genes, *ERG10* and _cyto_ *ETR1*, to construct the reverse-β-oxidation pathway, thereby enabling the conversion of acetyl-CoA into butyryl-CoA (Dellomonaco et al., 2011; Lian and Zhao 2015). To generate a complete butanol-production pathway, *Adhe2* or *BdhB* from *C. acetobutylicum* were heterologously expressed with or without *EutE* from *E. coli* (Figure 1A). Heterologous expression of these *C. acetobutylicum* genes increased butanol production more than heterologous expression of the indicated *Y. lipolytica* genes, even without codon optimization. When expressing heterologous genes, it is often expected that genes originating from yeasts or other eukaryotic cells will be expressed at higher levels in eukaryotic cells (such as *S. cerevisiae*). However, the distance in a phylogenetic tree does not seem to guarantee better performance, when expressed in a heterologous host.

The choice of the alcohol dehydrogenase used, either Adhe2 or BdhB, did not significantly affect the butanol titer. Although AdhE2 is a bi-functional enzyme that converts butyryl-CoA into butyraldehyde and then to n-butanol, it cannot fully support butanol production without *EutE* expression, suggesting that sufficient enzyme levels are required for each step in n-butanol synthesis. Of the strains expressing heterologous gene combinations, the XA-3 strain harboring the set comprised of *Hdb*, *Crt*, *EutE*, and *AdhE2* resulted in the highest butanol titer of 7.2 mg/L, followed by the XA-4 strain harboring the set comprised of *Hdb*, *Crt*, *EutE*, and *BdhB* (6.8 mg/L) (Figure 1B). Based on the similar butanol titer with a half size of *BdhB* compared to *Adhe2*, the butanol production pathway genes in the XA-4 strain was selected as the combination of choice for further experiments throughout this study.

**FIGURE 2:**
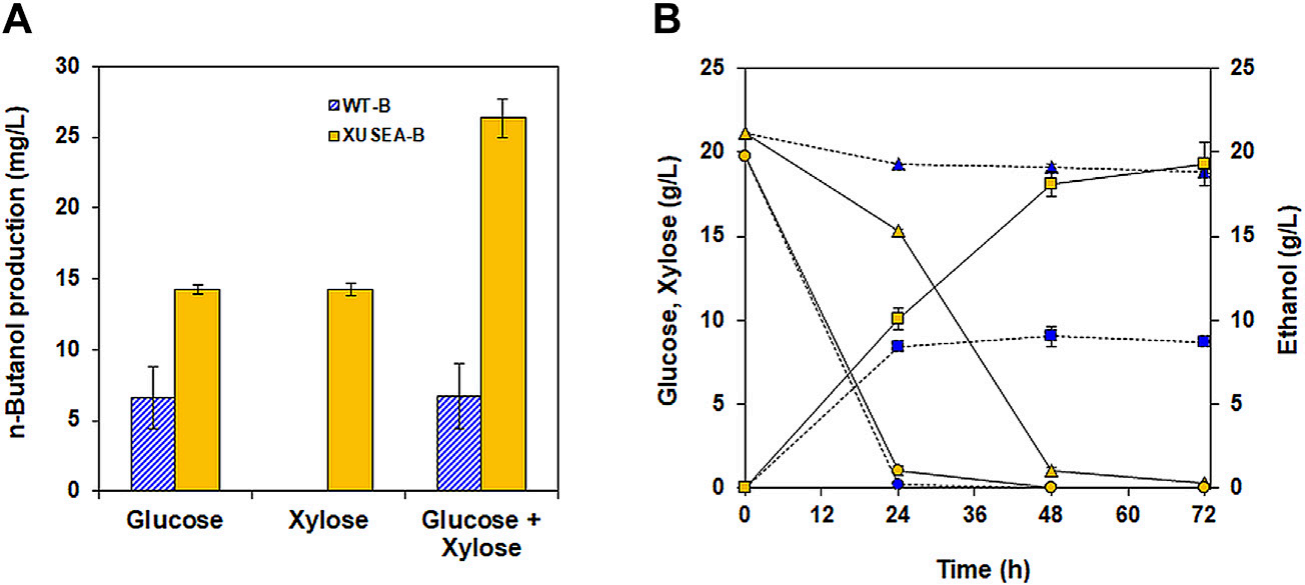
Comparison of n-butanol production between the WT-B (blue) and XUSEA-B (yellow) strains during glucose and/or xylose fermentation. Fermentation was conducted with an initial OD of 0.2. **(A)** n-butanol titer after 72 h of fermentation. **(B)** glucose (circles)/xylose (triangles) consumption and ethanol production (squares) during co-fermentation. The error bars represent the standard deviations obtained using biological triplicates.

The n-butanol production pathway with the selected genes were introduced into both a xylose utilizing strain of XUSEA and a wild-type strain of *S. cerevisiae* BY4741, generating XUSEA-B and WT-B, respectively. Then, the n-butanol production performance of XUSEA-B and WT-B was compared during glucose fermentation, xylose fermentation, and glucose/xylose co-fermentation (Figure 2). During glucose fermentation, the XUSEA-B strain showed 2.1-fold higher n-butanol production than the WT-B strain (14.2 mg/L and 6.6 mg/L, respectively; Figure 2A). The XUSEA-B strain produced the same amount of n-butanol during xylose fermentation, whereas the WT-B strain produced no n-butanol because it could not utilize xylose. During glucose/xylose co-fermentation, XUSEA-B produced 26.3 mg/L of n-butanol within 72 h, which was 3.9-fold higher than that produced by WT-B (6.7 mg/L; Figures 2C,D), suggesting that the xylose-utilizing strain could serve as a promising host for producing n-butanol, an acetyl-CoA-derived product.

### Transcriptomic Landscape Revealed the Redirected Carbon Flux for Improved n-Butanol Production in a Xylose-Utilizing Strain

**FIGURE 3:**
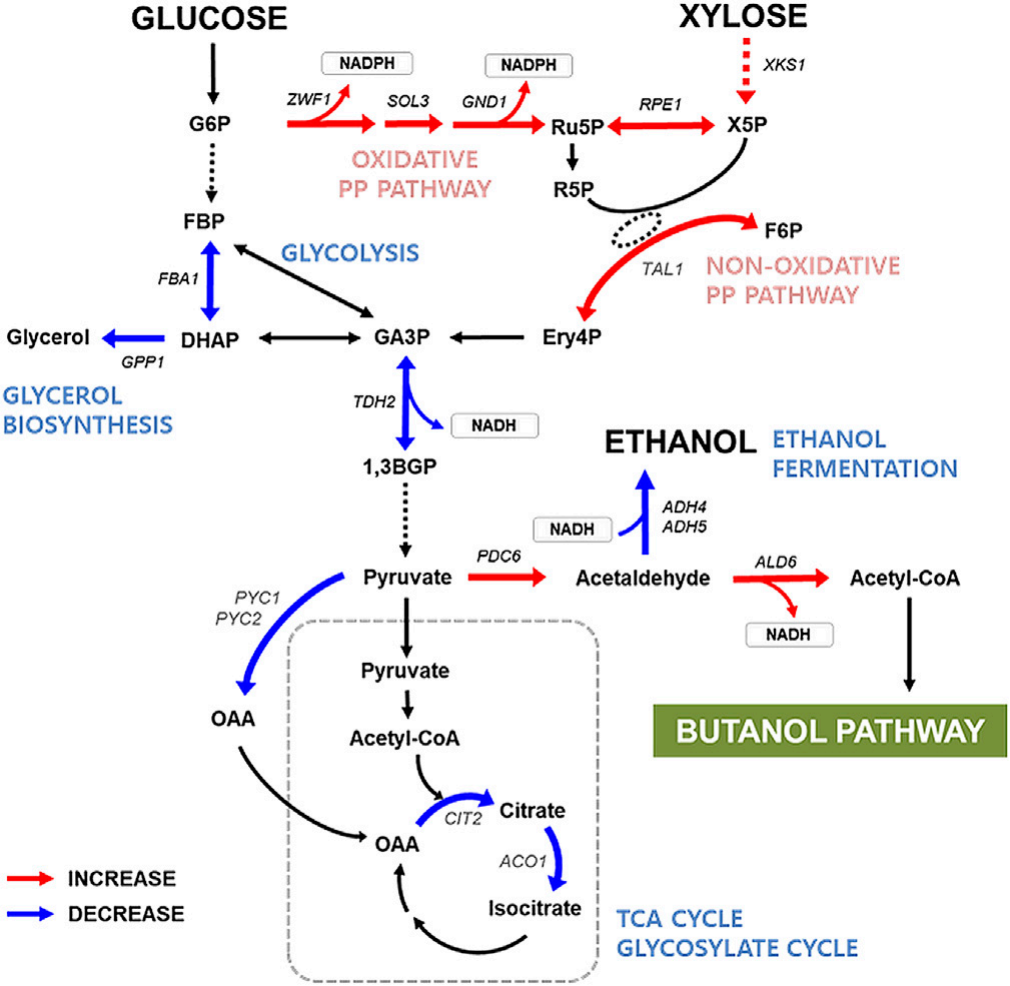
Transcription levels of the genes involved in central carbon metabolism in the XUSEA-B strain during glucose or glucose/xylose co-fermentation. Red/blue arrows indicate the enzymatic steps with more than 2-fold increased/decreased transcription levels in XUSEA-B compared to those in WT-B strain. The cells were grown on glucose medium (20 g/L glucose) or a mixed-sugar medium (20 g/L glucose and 20 g/L xylose) under micro-aerobic conditions. Sampling was conducted during the exponential phase of cell growth. The gene symbols were referenced from the *Saccharomyces* Genome Database.

To understand the mechanism underlying efficient n-butanol production by the xylose-utilizing strain, we analyzed the global transcript profiles of XUSEA-B and WT-B during glucose fermentation and glucose/xylose co-fermentation. Compared with WT-B, the XUSEA-B strain showed marked differences in the gene-expression landscape (170 upregulated and 84 downregulated genes with glucose fermentation versus 172 upregulated and 82 downregulated genes with mixed-sugar fermentation) (Figure 3). Transcriptional changes in genes associated with core carbon metabolism and linked with the butanol-production pathway were similar between both fermentation conditions, despite the use of different carbon sources. The XUSEA-B strain appeared to show elevated central carbon flux through the n-butanol-production pathway, resulting from pathway upregulation due to increased cofactor generation and reduced use of the acetyl-CoA precursor. The *ZWF1*, *SOL3*, and *GND1* genes (involved in the oxidative pentose phosphate pathway, from which the factor NADPH is generated), were upregulated in XUSEA-B compared to WT-B by 2.81-, 4.11-, and 7.82-fold during glucose fermentation and by 3.74-, 5.54-, and 9.93-fold during glucose/xylose co-fermentation, respectively (Figure 3, Table 3).

**TABLE 3:** Fold-changes in the expression levels of genes involved in metabolic pathways in XUSEA-B strain, versus the WT-B strain. G: glucose, GX: glucose and xylose.

Gene symbol	Expression fold-change		Function
	G	GX	
**Glycolysis**			
*TDH2*	0.42	0.40	Glyceraldehyde-3-phosphate dehydrogenase
*FBA1*	0.49	0.50	Fructose 1,6-bisphosphate aldolase
*EN O 2*	0.43	0.48	Phosphopyruvate hydratase
**Pentose phosphate pathway**			
*Oxidative branch*			
*ZWF1*	2.81	3.74	Glucose-6-phosphate dehydrogenase
*SOL3*	4.11	5.54	6-phosphogluconolactonase
*GND1*	7.82	9.93	6-phosphogluconate dehydrogenase
*Non-oxidative branch*			
*RPE1*	100	95.1	D-ribulose-5-phosphate 3-epimerase
*XKS1*	9.76	29.5	Xylulokinase
*TAL1*	3.15	3.96	Transaldolase
**Electron transport change**			
*CYC1*	0.36	0.48	Cytochrome c, isoform 1
*AAC3*	2.24	2.2	Mitochondrial inner membrane ADP/ATP translocator
**TCA cycle and glyoxylate cycle**			
*CIT2*	0.32	0.50	Citrate synthase
*ACO1*	0.27	0.49	Aconitase
*PYC1*	0.29	0.42	Cytoplasmic pyruvate carboxylase; decarboxylates pyruvate to oxaloacetate
*PYC2*	0.44	0.49	Cytoplasmic pyruvate carboxylase; decarboxylates pyruvate to oxaloacetate
**Ethanol fermentation**			
*ADH4*	0.1	0.09	Alcohol dehydrogenase isoenzyme IV
*ADH5*	0.39	0.43	Alcohol dehydrogenase isoenzyme V
*PDC6*	14.4	6.07	Pyruvate decarboxylase; decarboxylates pyruvate to acetaldehyde
*ALD6*	3.36	0.77	Cytosolic aldehyde dehydrogenase
**Glycerol biosynthesis and degradation**			
*GPP1*	0.34	0.27	Glycerol-1-phosphatase
*GCY1*	3.98	14	Glycerol dehydrogenase
**Amino acid biosynthesis**			
*ILV3*	0.28	0.39	Dihydroxy-acid dehydratase
*EEB1*	0.14	0.28	Medium-chain fatty acid ethyl ester synthase/esterase
*LEU1*	0.26	0.43	Isopropylmalate isomerase
*LEU2*	0.29	0.30	Beta-isopropylmalate dehydrogenase
*LEU9*	0.36	0.26	Alpha-isopropylmalate synthase I
*TRP2*	0.46	0.39	Anthranilate synthase
**Other functions**			
*OYE3*	7.16	12.21	NADPH oxidoreductase containing a flavin mononucleotide
*SER33*	3.88	3.44	3-phosphoglycerate dehydrogenase
*YEF1*	3.69	7.64	ATP-NADH kinase; phosphorylates both NAD and NADH
*ERG10*	0.32	0.27	Cytosolic acetyl-CoA C-acetyltransferase

It should be noted that genes involved in the non-oxidative pentose phosphate pathway (such as *RPE1* and *TAL1*) and *XKS1* were highly upregulated in the XUSEA-B strain, compared to WT-B (Figure 3, Table 3). These differences were mainly due to the genetic background XUSEA (the parental strain of XUSEA-B), in which the above-mentioned genes were overexpressed (Tran Nguyen Hoang et al., 2018; Hoang Nguyen Tran et al., 2020). However, it is noteworthy that *RPE1* mRNA expression was upregulated by approximately 100-fold in the XUSEA-B strain. This finding suggests that carbon flux was enhanced through glycerate-3-phosphate, an interconnection point between the oxidative and non-oxidative pentose phosphate pathways. The transcription of *PDC6*, which is involved in converting pyruvate to acetaldehyde, was also strongly upregulated suggesting that the acetyl-CoA was increased in XUSEA-B. In contrast, the *FBA1* and *TDH2* genes (involved in glycolysis), were down-regulated more than 2-fold in XUSEA-B, leading to a low carbon flux through central carbon metabolism via glycolysis.

### Supplementation With Acetate Improved n-Butanol Production by *S. cerevisiae*

**FIGURE 4:**
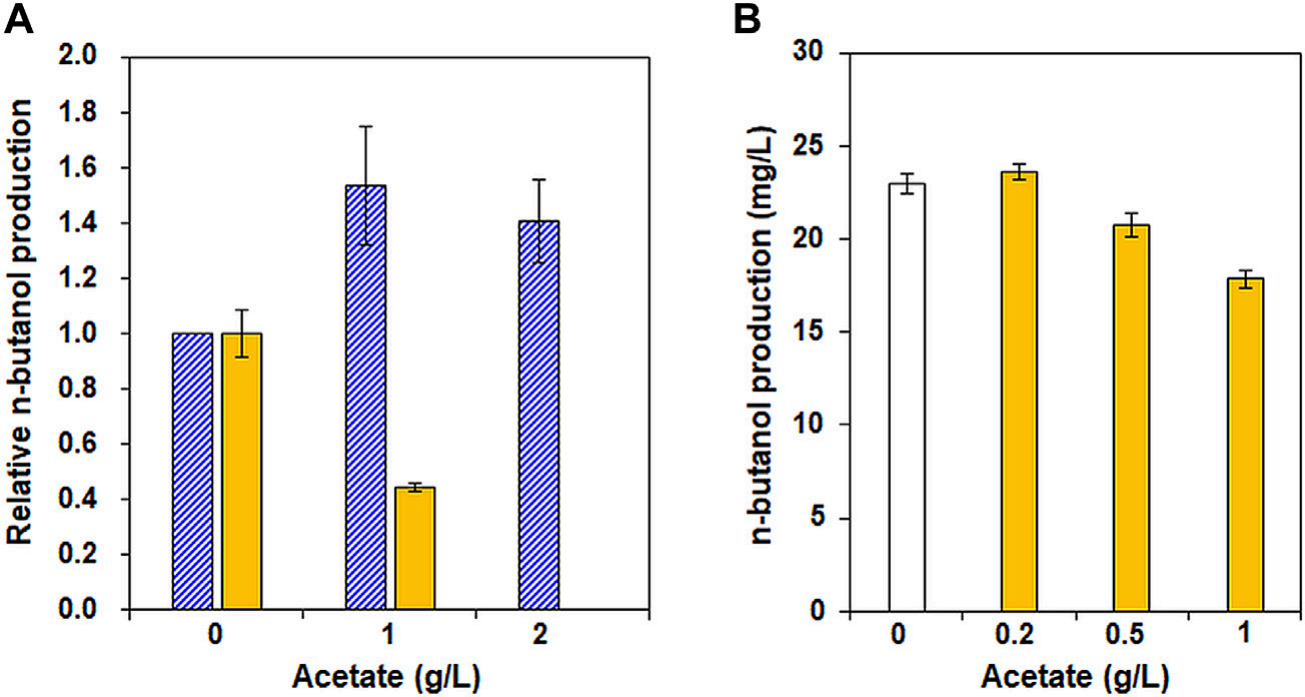
Effect of acetate supplementation on n-butanol production by XUSEA-B strains during glucose and/or xylose fermentation. Fermentation was conducted with an initial OD of 0.2 and various concentrations of acetate (0, 1, or 2 g/L). **(A)** n-butanol production during glucose (pattern, blue) or xylose (solid, yellow) fermentation. **(B)** n-butanol production during glucose/xylose co-fermentation without (white) and with (yellow) acetate supplementation (0.2 g/L, 0.5 g/L, or 1 g/L). The error bars represent standard deviations obtained using biological triplicates.

After confirming the positive effect of increased acetyl-CoA availability in XUSEA-B, based on transcriptomic analysis, the effect of acetate supplementation on n-butanol production was investigated as a strategy for supplying additional acetyl-CoA. To this end, n-butanol production by XUSEA-B was evaluated during glucose and/or xylose fermentation supplemented with various concentrations of acetate (0 g/L, 1 g/L, or 2 g/L). During glucose fermentation, acetate supplementation increased n-butanol production by XUSEA-B (Figure 4). XUSEA-B strains produced 40% or 28% more n-butanol in the presence of one or 2 g/L acetate, respectively. In contrast, acetate supplementation did not result in increased n-butanol production during xylose fermentation. The production of n-butanol decreased by 56% in the presence of 1 g/L acetate during 48 h of xylose fermentation. No n-butanol was produced in the presence of 2 g/L acetate in the medium because the cell growth was significantly inhibited (data not shown). Interestingly, the negative effect of acetate supplementation on n-butanol production during xylose fermentation was compensated in the presence of glucose. During glucose/xylose co-fermentation, n-butanol production was reduced by 23% in the presence of 1 g/L acetate (Figure 4B).

**FIGURE 5:**
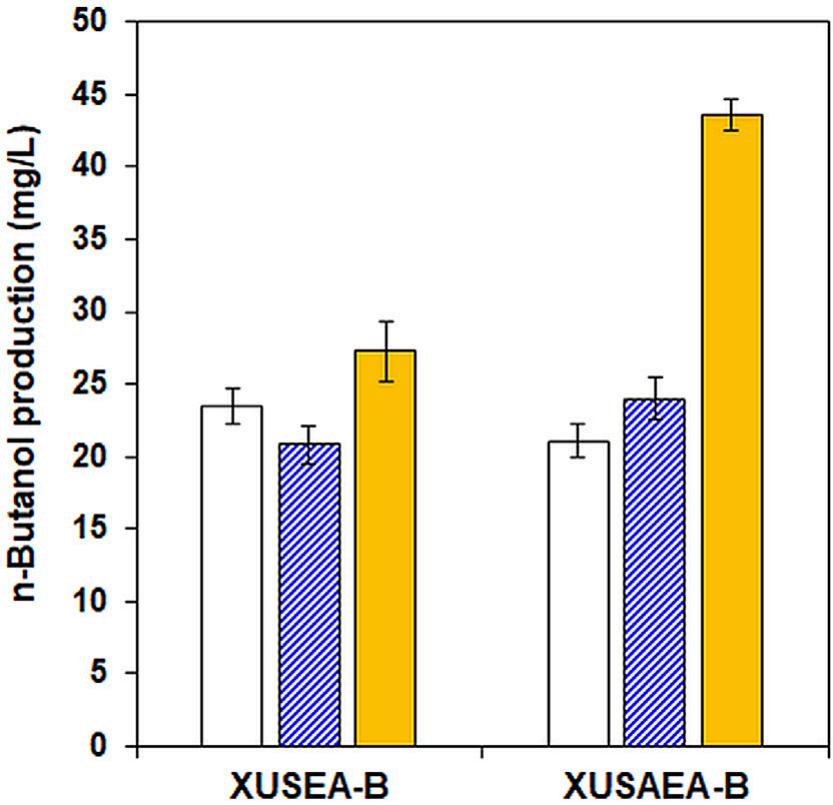
Comparison of n-butanol production by the XUSEA-B strain and the acetate-tolerant strain of XUSAEA-B during glucose/xylose co-fermentation. Fermentation was conducted using an initial OD of 0.2 with 0 g/L (solid, white) and 1 g/L (pattern, blue) of acetate supplementation at pH 5.0. n-butanol production at pH 6.5 was also evaluated with 1 g/L of acetate supplementation (solid, yellow). The error bars represent standard deviations obtained using biological triplicates.

Given the both positive and negative effects of acetate supplementation on n-butanol production, we introduced the n-butanol-production pathway into an acetate-tolerant glucose/xylose co-fermenting strain of XUSAE-57 (Ko et al., 2019). To promote the conversion of acetate into acetyl-CoA, a heterologous acetate-utilization pathway was additionally integrated by overexpressing a mutant version of ACS^*L641P*^ from *Salmonella enterica* and AADH from *E. coli*, thereby generating XUSAEA-B. The XUSAEA-B, an acetate tolerant and utilizing strain, showed dramatically improved n-butanol production resulting in an n-butanol titer of 46.5 mg/L with acetate supplementation (1 g/L) during glucose/xylose fermentation at pH 6.5, under which condition the inhibitory effect of acetate has been shown to decrease (Figure 5).

### Cellulosic n-Butanol Production Using Glucose/Xylose Co-fermenting *S. cerevisiae*

**FIGURE 6:**
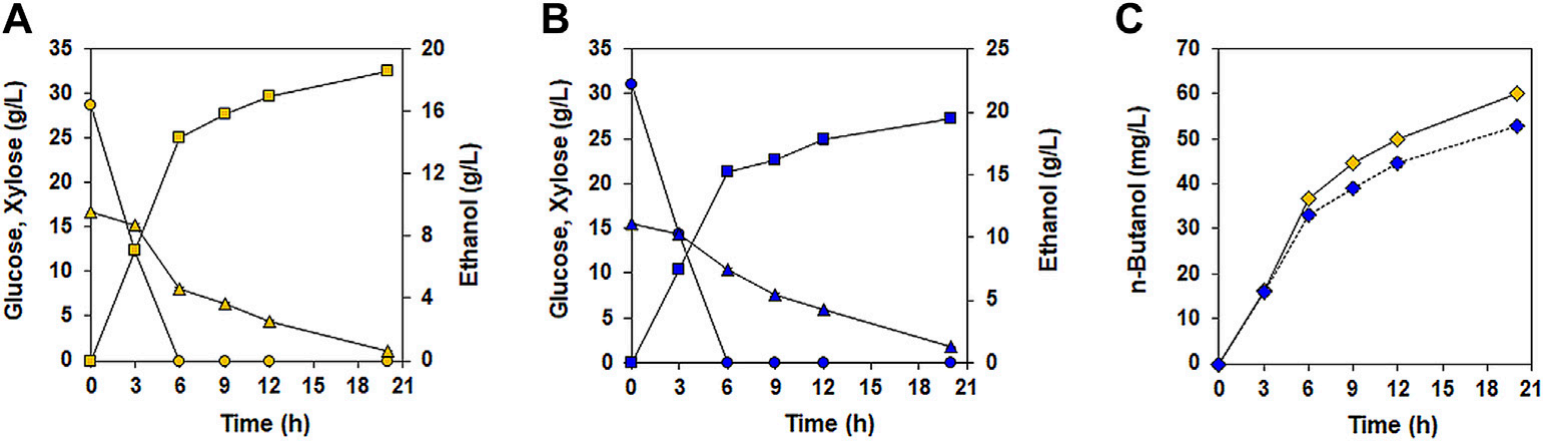
n-butanol fermentation by the XUSAEA-B strain using lignocellulosic hydrolysates. Fermentation was conducted with *Miscanthus* hydrolysates pretreated with diluted acids. Fermentation with YSC medium composed of the same concentration of sugars (30 g/L of glucose and 15 g/L of xylose) was also conducted under the same conditions. The initial OD and pH were 15 and 6.5, respectively. **(A)** Fermentation performance using lignocellulosic hydrolysates (yellow). **(B)** Fermentation performance using YSC medium (blue). **(C)** n-butanol production during fermentation using lignocellulosic hydrolysates (solid line, yellow diamond) and synthetic medium (dash line, blue diamond). Ethanol production (squares) and glucose (circles) and xylose (triangles) consumptions were measured during fermentation. The error bars represent standard deviations obtained using biological triplicates.

Encouraged by the increased n-butanol production during glucose/xylose co-fermentation in the presence of acetate, we evaluated the n-butanol-production performance of XUSAEA-B from lignocellulosic hydrolysates of *Miscanthus sacchariflorus Goedae-Uksae*, which were prepared through a H_2_SO_4_-catalyzed hydrothermal process (Figure 6). During a 21 h fermentation, XUSAEA-B completely utilized glucose and xylose, and produced 60.1 mg/L of n-butanol (Figures 6A,C). Interestingly, n-butanol production from lignocellulosic hydrolysates was 14% higher than that from YSC medium which was supplemented with same glucose and xylose concentrations as *Micanthus* hydrolysate (60.1 mg/L and 52.8 mg/L for lignocellulosic hydrolysates and YSC medium, respectively) (Figure 6C). To our knowledge, this study demonstrates n-butanol production from lignocellulosic hydrolysate by using a glucose/xylose co-fermenting *S. cerevisiae* for the first time whereas other previous studies mainly showed n-butanol production from synthetic glucose media (Lian et al., 2014; Sakuragi et al., 2015; Swidah et al., 2015; Schadeweg and Boles 2016a; Schadeweg and Boles, 2016b).

## Discussion

Yeast engineering for lignocellulosic biorefinery has primarily focused on either the expansion of substrate ranges or product profiles through individual approaches. However, the product profiles of *S. cerevisiae* capable of xylose utilization are limited to only a few compounds, including isobutanol, carotene, and 1-hexadecanol (Brat and Boles, 2013; Guo et al., 2016; Sun et al., 2019). Recent reports highlighting the prospect of engineering a xylose-utilizing strain as a promising host for the production of acetyl-coA derived products (Kwak and Jin, 2017) offer easier diversion of product profiles from ethanol to other products, for a glucose/xylose co-utilizing *S. cerevisiae*.

In this study, we demonstrated the expansion of product profiles of a glucose/xylose co-fermenting *S. cerevisiae* to include the acetyl-coA derived product, n-butanol, which is a short chain alcohol similar to ethanol, however, has only been produced by *S. cerevisiae* from glucose with limited success. Despite the numerous attempts to develop n-butanol producing *S. cerevisiae*, the production titer remained suboptimal even with extensive metabolic engineering. There are several benefits associated with using a glucose/xylose co-fermenting strain for n-butanol production. For instance, it has a higher metabolic flux through acetyl-CoA compared to strains capable of only utilizing glucose (Kwak and Jin, 2017; Li et al., 2019). Additionally, it has an associated higher conversion yield per unit of biomass. These benefits were demonstrated in the current study. Specifically, the glucose/xylose co-fermenting *S. cerevisiae* strain, XUSEA-B, showed 3.9-fold higher n-butanol titer compared to the wild-type strain capable of only utilizing glucose during glucose/xylose co-fermentation. Given that the amount of xylose present was nearly half that of glucose, the additional n-butanol produced by XUSEA-B demonstrates a synergistic effect with an additional carbon source (xylose) in hydrolysates and an increased acetyl-CoA availability, supported by xylose catabolism in XUSEA-B.

In the study, we pointed out the increased acetyl-CoA pool of a glucose/xylose co-fermenting strain through analysis of the transcriptomic landscape in XUSEA-B, in which the increased metabolic flux was expected toward pyruvate, acetaldehyde, acetate, and cytosolic acetyl-CoA by re-directing the carbon flux through competing pathways such as the tricarboxylic acid (TCA) cycle, glyoxylate cycle, amino acid biosynthesis, and ethanol production (Figure 3, Table 1). The metabolic flux through pyruvate, specifically, could be directed toward acetaldehyde based on the highly upregulated expression of *PDC6* accompanied by downregulation of genes associated with pyruvate carboxylation, and those involved in the TCA cycle, glyoxylate cycle and amino acid biosynthesis employing pyruvate as a precursor. Moreover, the fate of acetaldehyde through the ethanol-fermentation pathway also appeared to be redirected toward acetate formation, based on the decreased expression of alcohol dehydrogenases encoded by *ADH4* and *ADH5* and increased expression of aldehyde dehydrogenase encoded by *ALD6* (Figure 3, Table 3) in XUSEA-B. Meanwhile, the high availability of the cytosolic acetyl-CoA contributed substantially to the improved n-butanol production in XUSEA-B during glucose and glucose/xylose fermentation, thus demonstrating the advantage of employing xylose-utilizing *S. cerevisiae* for the production of acetyl-CoA-derived biofuels and biochemicals from lignocellulosic biomass.

In XUSEA-B, the oxidative pentose phosphate pathway, a major route for NADPH production (Stincone et al., 2015), appeared to be actively regulated via upregulation of ZWF1, SOL3, and GND1, possibly supporting the notion that improved NADPH availability improves metabolic flux through the NADPH-dependent rate-limiting step in the n-butanol production pathway. Indeed, the n-butanol pathway in the glucose/xylose co-fermenting strain, containing an NADPH-preferring cytoETR1 protein, was better supported by the sufficient NADPH cofactor present in the XUSEA-B strain.

In terms of glycerol-metabolism, expression of the glycerol-1-phosphatase gene (*GPP1*), which is important for glycerol biosynthesis, decreased (Figure 3, Table 3), whereas transcription of the glycerol dehydrogenase gene (*GCY1*), which participates in glycerol catabolism under micro-aerobic conditions, was increased. Similarly, suppressed glycerol synthesis combined with disruption of the ethanol-fermentation pathway reportedly stimulates n-butanol production (Lian et al., 2014; Swidah et al., 2015; Schadeweg and Boles, 2016b) by increasing the abundance of NADH, a driving force of the n-butanol pathway (Kim et al., 2015; Schadeweg and Boles, 2016a). Furthermore, the upregulation of genes related to cofactor regeneration, such as *OYE3*, *SER33*, and *YEF1*, suggest that XUSEA-B maintained an optimal balance by varying redox cofactors, not only for efficient n-butanol production. Understanding the transcriptomic characteristics of XUSEA-B strain not only help to explain the improved butanol production during glucose/xylose fermentation, but also could offer engineering strategies to further improve the butanol titer in this minimally engineered strain of XUSEA-B. These engineering targets could also be used for rewiring biosynthetic routes to produce acetyl-CoA-derived chemicals in the context of lignocellulosic biorefinery.

Previously, acetate supplementation increased n-butanol production in a native producer of *Clostridium sp.* by upregulating the expression levels of CoA-transferase genes, thereby increasing the availability of acetyl-CoA and butyryl-CoA, two main precursors of the n-butanol synthesis pathway (Chen and Blaschek, 1999). During lignocellulosic fermentation by *S. cerevisiae*, however, acetate is regarded as a major inhibitory compound impeding cell growth and sugar utilization rate, and thus limits the fermentation performance of *S. cerevisiae* (Helle et al., 2003; Jönsson et al., 2013), particularly during xylose fermentation (Ko et al., 2015). *S. cerevisiae* endogenously expresses *ACS1* and *ACS2*, which encode acetyl-coA synthetases to convert acetate into acetyl-CoA using ATP (van den Berg et al., 1996). Therefore, acetate supplementation could be expected to support additional carbon flux through the n-butanol biosynthetic pathway by enhancing acetyl-CoA, a crucial precursor for n-butanol production. As expected, in the current study n-butanol production increased during glucose fermentation in the presence of acetate. This could be explained due to the increased acetyl-CoA pool through the conversion of acetate by native *ACS* genes. The sufficient intracellular ATP supplied from glucose could also have alleviated the inhibitory effect of acetate (Zhang et al., 2016). During xylose fermentation, however, acetate supplementation did not positively affect n-butanol production, possibly due to insufficient detoxification and limited ATP generation by xylose (Casey et al., 2010). In fact, increased acetate concentration caused a decrease in the n-butanol titer during xylose fermentation (Figure 4A). Several strategies have been proposed to overcome the toxic effects of acetic acid while maintaining fermentation performance in *S. cerevisiae*. Ko *et al.* improved acetate tolerance of a xylose fermenting *S. cerevisiae* through adaptive laboratory evolution (Ko et al., 2019). Zhang *et al.* have coupled an acetate reduction pathway with xylose catabolism during cellulosic fermentation to improve sugar-to-product conversion yield while converting acetate into less inhibitory products (Zhang et al., 2016). When an acetate-tolerant glucose/xylose co-fermenting strain harboring an acetate conversion pathway, XUSAEA-B, was used, the beneficial effects of acetate supplementation were recovered resulting in significantly improved n-butanol production (Figure 5). Moreover, during lignocellulosic fermentation, XUSAEA-B produced 60.1 mg/L of n-butanol, accounting for a 14% higher yield compared to that obtained during fermentation using synthetic media with the same concentrations of sugars (52.8 mg/L), possibly due to the positive effect of acetate on elevating acetyl-CoA for the n-butanol pathway in lignocellulosic hydrolysates.

## Conclusion

In this study, we investigated the production of the acetyl-coA-derived product, n-butanol, by a glucose/xylose co-fermenting *S. cerevisiae* strain. The transcription profiles of *S. cerevisiae* with efficient xylose catabolism revealed a modified cellular network that better supported the generation of acetyl-CoA and cofactors required for n-butanol fermentation. Incorporating acetate catabolism further improved n-butanol production from lignocellulosic hydrolysates. Consequently, the results of this study show the potential of using a glucose/xylose co-fermenting strain and lignocellulosic biomass as more attractive production host and resource for biorefinery.

## Data Availability

The original contributions presented in the study are included in the article/Supplementary material, further inquiries can be directed to the corresponding author.
