# Supplementary figures and images for: Glucose/Xylose Co-Fermenting Saccharomyces cerevisiae Increases the Production of Acetyl-CoA Derived n-Butanol From Lignocellulosic Biomass
Source: Front Bioeng Biotechnol. 2022 Feb 16;10:826787. doi: 10.3389/fbioe.2022.826787 (PMC8889018; doi:10.3389/fbioe.2022.826787)

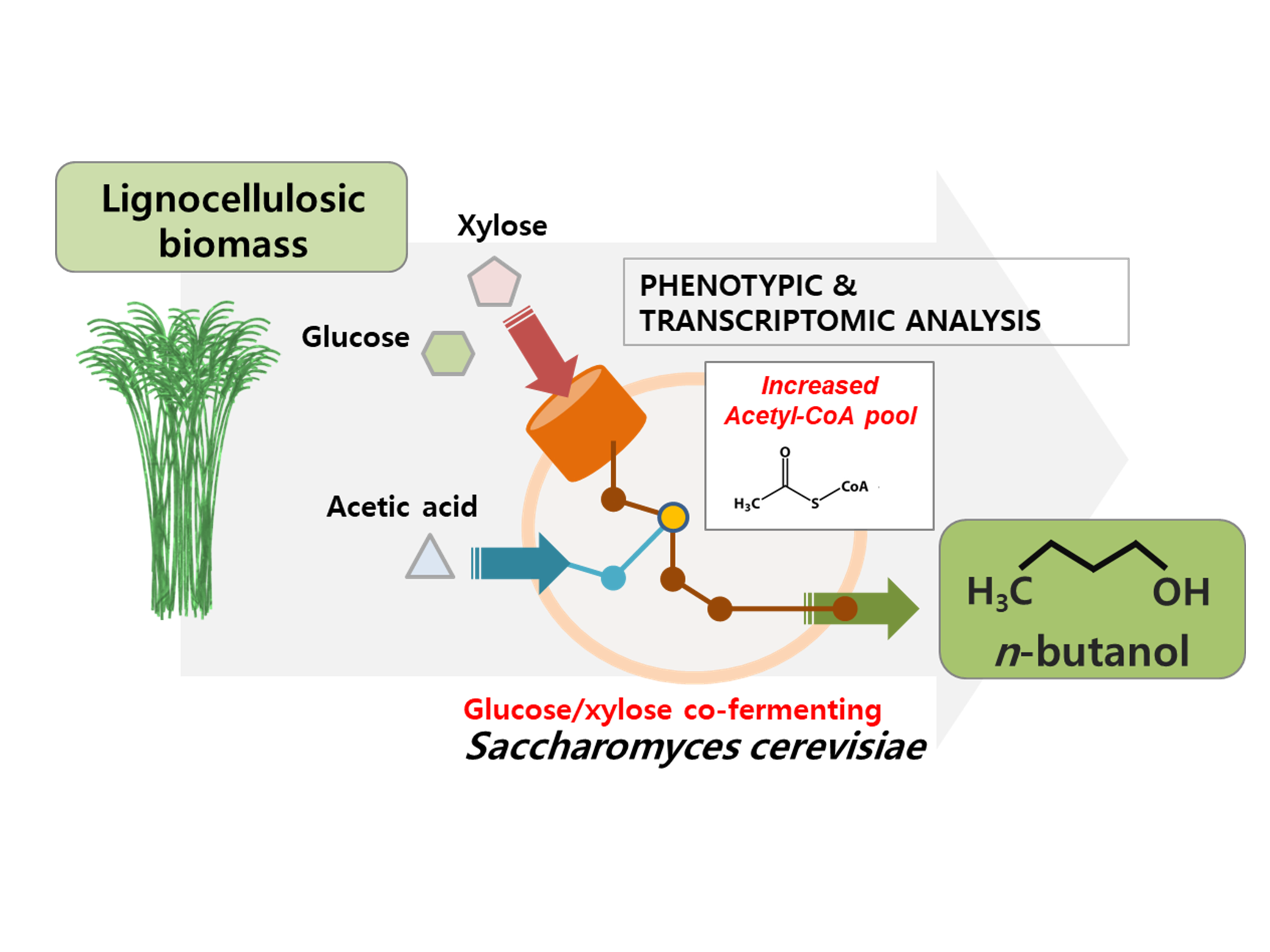

Supplement: Supplementary file 1 [file Image1.TIF]
